# Network approach in health behavior research: how can we explore new questions?

**DOI:** 10.1080/21642850.2019.1682587

**Published:** 2019-11-05

**Authors:** Samvel Mkhitaryan, Rik Crutzen, Esther Steenaart, Nanne K. de Vries

**Affiliations:** Department of Health Promotion, CAPHRI Maastricht University, Maastricht, The Netherlands

**Keywords:** Belief networks, network analysis, psychological networks

## Abstract

**Background:** The network approach has recently been introduced to clinical psychology and provides a powerful framework for analyzing variables in a system. Since then, its applications have rapidly spread to various fields of social sciences. Unlike in the case of clinical psychology, the peculiarities of the phenomena under study in social sciences have not received sufficient attention. In this paper, along with practical illustrations, we discuss what a system of psychological variables represents and what the interrelationships between the variables mean in the context of health behavior research. Additionally, we explore the structural analysis of the system which has not been the focus of the recent applications of network analysis in health psychology.

**Discussion:** In this paper, we illustrate two approaches of incorporating observable behavioral variables in a system and strategies for investigating structural components of the system. We illustrate these two approaches with an analysis of cross-sectional data on adolescents’ beliefs and behavior with respect to registering their choice regarding organ donation in the Netherlands. Furthermore, with this paper, we wish to facilitate a larger discussion on conceptualizing networks of psychological variables, which will guide the analysis and the interpretation of node level interactions as well as network level structures.

## Introduction

Theories in health behavior^1^ research, akin to other disciplines of social sciences, attempt to explain phenomena of interest (e.g. health behavior) by describing components (e.g. psychosocial variables) related to such phenomena, as well as the relationship between these components. Psychological variables that are intervention targets, are oftentimes represented as latent constructs or composite variables (e.g. self-efficacy, attitude), measured by observable indicators (e.g. self-reported items in questionnaires, reaction times), that explain the variation in the outcome of interest (e.g. health behavior). The latent constructs and composite variables are higher level abstractions (Peters & Crutzen, 2018), which are created by either formative or reflective models (Schmittmann et al., 2013) because they are impossible to observe (i.e. always require measurement by means of, for example, questionnaires, reaction times). The *reflective* models view item-scores as reflecting one’s position on the underlying latent construct (i.e. the latent construct causes the item-scores). As opposed to the reflective models, the *formative* models assume that the item-scores create the construct without hypothesizing that the latent construct exists independently of the observable indicators (Gruijters & Fleuren, 2018).

The appropriateness of reflective and formative approaches has recently been debated from a psychometric perspective (Borsboom, Cramer, & Kalis, 2018; Dalege et al., 2016; Gruijters & Fleuren, 2018; Hevey, 2018; Peters & Crutzen, 2018; Schmittmann et al., 2013) as well as from a pragmatic stance (Peters & Crutzen, 2017). From a psychometric perspective, Schmittmann et al. (2013) argue that particular assumptions of causal relationships between the construct and the respective indicator variables are problematic for psychological testing. For example, while causal relations between indicator variables are inconsequential in a formative model, the reflective model does not allow causal interrelationships between the indicator variables (i.e. assumes local independence) (Schmittmann et al., 2013). However, such assumptions may be incorrect. For instance, in an experimental study (Sniehotta, 2009), intervening on normative beliefs had a positive effect on perceived behavioral control. Such direct causal interrelationships between the lower level determinants are in direct contradiction with the assumption of local independence in reflective models. Moreover, these interrelationships are not inconsequential – instead, they carry important information on the existence of possible self-reinforcing processes. Similar arguments are made about constructs in psychopathology (e.g. depression) (Borsboom et al., 2018).

From a pragmatic stance, one could argue that latent constructs or composite variables may obscure important interrelationships between observable indicators that are of practical importance (as shown in the example above). Moreover, it is reasonable to assume that not all indicator variables are equally important in relation to the outcome of interest – there are presumably more central and less central variables. However, latent or composite variables do not convey such information and, as a consequence, the targets for behavior change interventions become less specific. In this regard, Peters and Crutzen (2017) view such constructs as useful metaphors rather than actual entities that exist in our mind. They further argue that representing psychological variables as emergent phenomena with a practical level of specificity is of great importance because these ‘specific’ variables are the ones that behavior change interventions target (Peters & Crutzen, 2017). Additionally, focus on the lower level determinants may help interventionists capture changes that would otherwise be masked (Peters & Crutzen, 2017).

Recently, there were multiple attempts to represent psychosocial constructs as emergent phenomena in a network of causally interacting variables (Brandt, Sibley, & Osborne, 2018; Dalege et al., 2016; Hevey, 2018; Rucci et al., 2018; Schmittmann et al., 2013; van Zyl, 2018). Seeing psychological variables as emergent phenomena means that the higher-level construct (e.g. self-efficacy) emerges from a constellation of lower level determinants (e.g. specific self-efficacy beliefs). The emergence of these higher-level constructs implies that the lower level determinants self-organize into hierarchical structures and form a system that has a ‘behavior’ that is more than the ‘behaviors’ of its constituent parts (i.e. lower-level determinants).

In contrast^2^ to the latent variable approach, the network approach represents observable indicators as nodes in a network and looks at the interrelationships between them without postulating the existence of latent constructs. Consequently, researchers can explore the determinants of behavior and evaluate the relative importance of each specific variable in a system. Moreover, researchers can explore how the networks structurally differ given the presence or absence of the outcome of interest. In available publications and tutorials, there is lack of discussion on what the system or network^3^ of psychological variables and causal interrelationships between them mean or represent. The authors mostly looked at the representation of nodes and explored node level measures such as betweenness, closeness and degree centrality (Brandt et al., 2018; Dalege et al., 2016; Epskamp, Borsboom, & Fried, 2018; Hevey, 2018; van Zyl, 2018). Although such analyses give important insights into the most important variables in a given network, they do not express much about the meaning of the overall network. In addition, we noticed that the overall structure of the network has not been a focus of interest in previous research on networks of psychological variables. However, as we argue in this paper, it may be of equal importance to investigate network topology of a behavior of interest. Descriptive and statistical comparisons of networks between groups with the presence or absence of the outcome of interest may provide insights into the structural predictors of the outcome of interest and thus inform future interventions.

In the present paper, we aim to contribute to the recent efforts to introduce the network approach to health behavior research (Hevey, 2018; Rucci et al., 2018; van Zyl, 2018) by discussing a strategy for incorporating the outcome of interest in network analysis and for investigating network topologies between groups that do or do not exhibit an outcome of interest. We begin by describing what the network approach is and what questions we can explore. Subsequently, we discuss two approaches of incorporating the behavior of interest in the network analysis.

## Network approach: what questions can we explore?

The network approach overcomes the unrealistic assumptions of reflective and formative models and addresses the pragmatic issues discussed above. The network perspective views the phenomenon of interest (e.g. depression, attitudes) as emergent in a system of interacting variables. Emergence of the phenomenon in a system means that a particular constellation of variables ‘defines’ that phenomenon. This particular constellation of variables that define the phenomena refers to the structure of the system and the dynamics of interactions between the system components. For example, Borsboom and Cramer (2013) represent different psychopathologies as emerging from a network of interrelated symptoms. This way, the outcome variables (e.g. anxiety, depression), that are often latent constructs, are represented as emerging from a network of their constituent observable indictors (e.g. symptoms). By taking such a mereological stance, researchers investigate the distinct features of these constellations (i.e. network structure) and the relative importance of their constituent components.

The transfer of network analysis from clinical psychology to health behavior research requires a discussion on the peculiarities of the phenomena under study. For example, health behavior research is often concerned with explaining behaviors (e.g. smoking, physical activity) that are observable rather than latent. Consequently, the question arises whether we shall consider the phenomenon of interest as resulting from a system or we shall conceive it as a constituent of a system. Another important question pertains to the causal interrelationships between the items – while symptoms can cause one another (e.g. fatigue causes irritation and vice versa) it is yet to be discussed how causal interactions apply to psychological determinants of health behaviors (e.g. subjective norms and attitudes). In either case, one ought to conceptualize what the system is in order to decide what the constituents of the system are or should be. We explore these and other questions sequentially in this paper with examples of an analysis^4^ of cross-sectional data on adolescents’ beliefs and behavior with respect to registering the choice regarding organ donation in the Netherlands (more information on the data can be found in Steenaart, Crutzen, and de Vries (2018)).

Before we begin with the statistical analyses, we ought to conceptualize the network that we aim to formulate. More specifically, we need to answer the following questions: What phenomena do we conceive as emergent and where do they emerge from? When the phenomenon of interest (e.g. self-efficacy) is unobservable, we can follow the network approach as it is currently applied in clinical psychology and regard that phenomenon as emerging from a system of its constituent observable indictors (e.g. items). However, how do we conceptualize an outcome of interest in a network framework when the outcome of interest is observable (e.g. behavior)? In this regard, there are two possible scenarios. In the first scenario, one may regard the observable behavior as a component of a system and subsequently investigate how different lower level determinants relate to it and to one another. In the second scenario, one may regard the behavior as emerging from ‘causal’ interactions between these determinants. Such distinction may not be immediately clear or mutually exclusive but we hope to illustrate that each of these scenarios allows investigation of different research questions and implies different analytical strategies. For example, the former may be used for exploring learning processes and behavioral feedback loops, while the latter allows exploration of structural components unique to a particular behavior.

## Behavior as a constituent in a system

Recent applications of the network approach to psychological variables treated observable behavioral variables as nodes in a system and explored the ways in which other psychological variables relate to the outcome variable and to each other (Brandt et al., 2018; Rucci et al., 2018). In such cases, while the latent constructs that represent different psychological domains can be viewed as emerging from them, the behavior itself is not emergent in a system but rather a component in the system. In a network framework, the components in the system are hypothesized to be engaged in ‘causal’ relationships with each other.

In order to facilitate a meaningful understanding and interpretation of the system, it is imperative to conceptualize what causal relationships mean between a set of psychological and behavioral variables. One way of doing so is to invoke theories of spreading activation from the literature on social cognition (Gawronski & Payne, 2010). We can look at psychological variables such as attitudes and intentions as mental representations that emerge in a network of respective beliefs. The corresponding beliefs then do not cause each other in a literal sense but rather cause each other’s emergence in the focal memory on which cognitive processes operate (discussed more in the subsequent sections). In other words, the relationships between beliefs are probabilistic rather than deterministic. The mutual reinforcement of concepts in the focal memory may stimulate one to engage in a certain behavior. The action then is implemented once a certain amount of stimulation is accumulated in the behavior (i.e. a certain threshold is achieved in the behavioral variable) (Gawronski & Payne, 2010).

**Figure 1.**
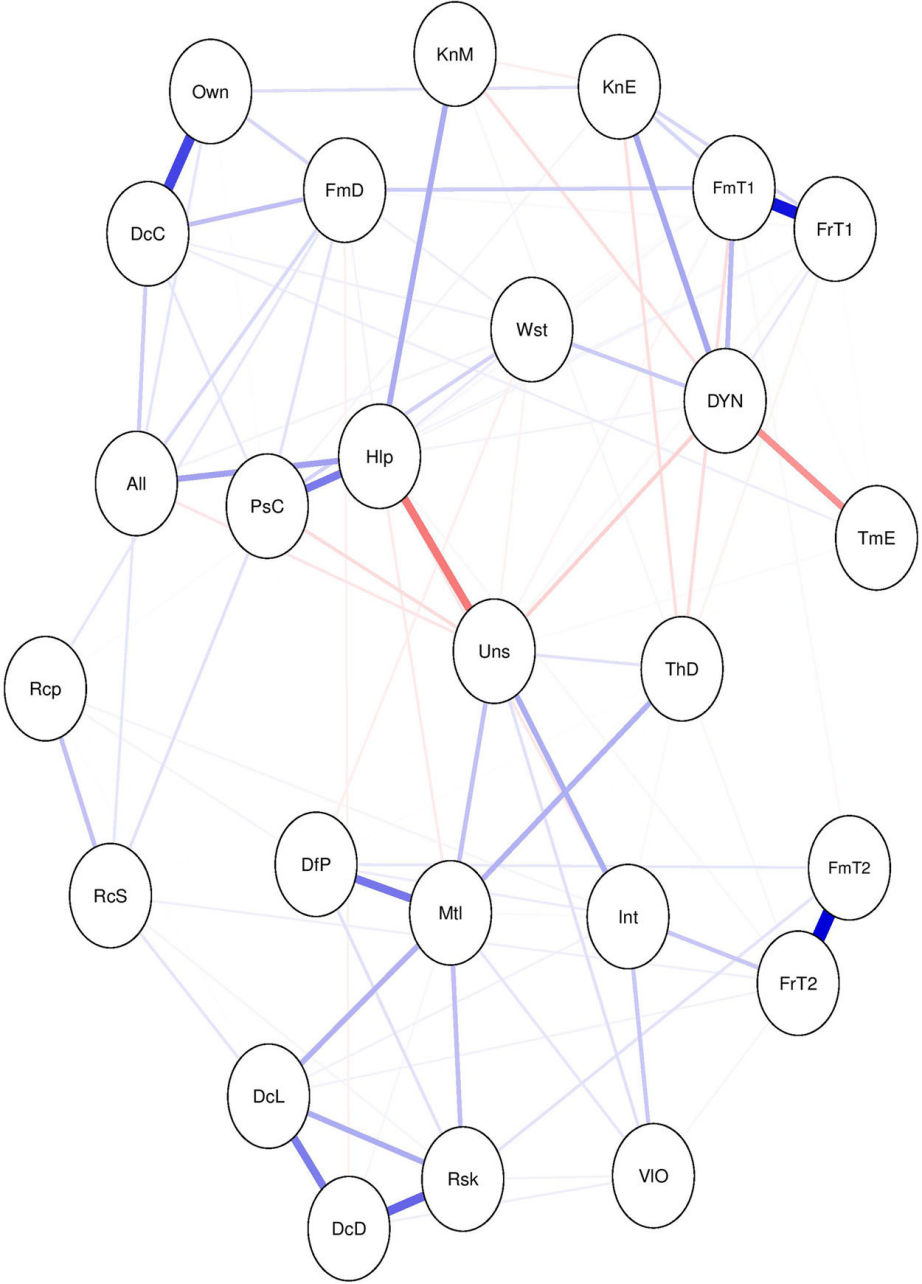
A network of items on beliefs about organ donation and donor registration status (DYN). Note: The list of items and the corresponding abbreviations are presented in the supplementary materials.

The inclusion of the observed behavioral variable in the network as a node in a system may be interesting for exploring direct as well as indirect relationships between the predictor variables and the outcome of interest. To illustrate this point, consider the decision to register as an organ donor in a network of items that represent beliefs about organ donation (see Figure 1).

In Figure 1, we may examine the interrelationships between items (lines between the nodes), the direction (negative relationships represented by red lines and positive relationships in blue) and the strength of these relationships (the width of the line). For example, being an organ donor (DYN) is positively associated with one’s belief of knowing enough (KnE) and feeling comfortable in talking to family members about organ donation (FmT1). The association between ‘knowing enough’ and ‘being registered as an organ donor’ is adjusted for the rest of the associations in the network.

**Figure 2.**
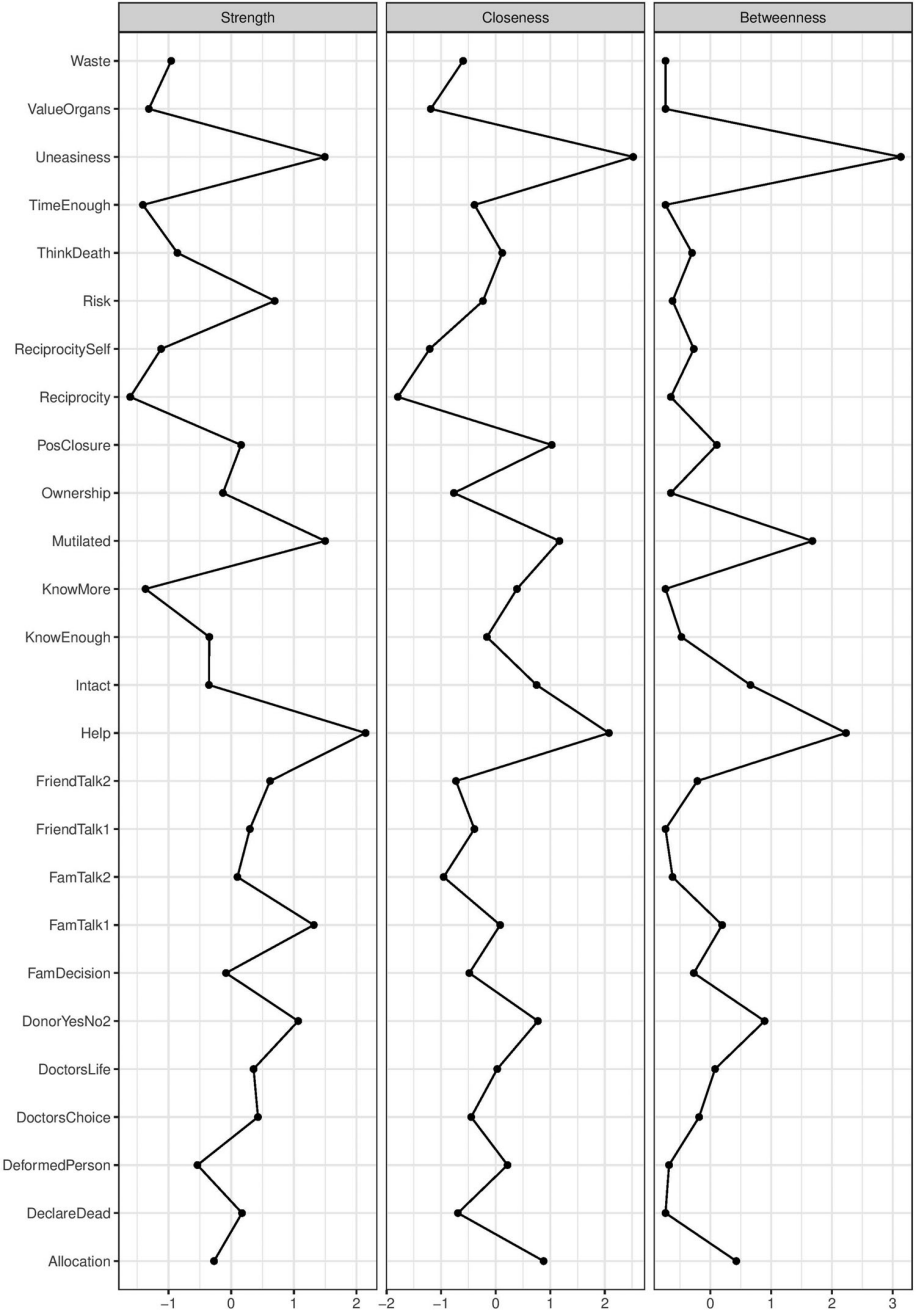
Centrality scores for all nodes in the network.

In addition to direct associations, we can examine the indirect associations (predictive mediations) between the variables. For example, the concern of mutilation (Mtl) indirectly relates to registration status through not wanting to think about death (ThD). The indirect relationships represent predictive mediations between variables (Epskamp & Fried, 2018). In addition to exploring the direct and indirect relationships between the predictors and the outcome of interest, researchers attempt to investigate the relative importance of each variable by calculating centrality scores (e.g. strength, betweenness) for each variable and the shortest paths to a focal variable (e.g. by applying Dijkstra’s algorithm) (Brandt et al., 2018; Hevey, 2018; Rucci et al., 2018). To illustrate this, we calculated three centrality measures for each node, namely, strength, closeness and betweenness centrality (see Figure 2).

**Figure 3.**
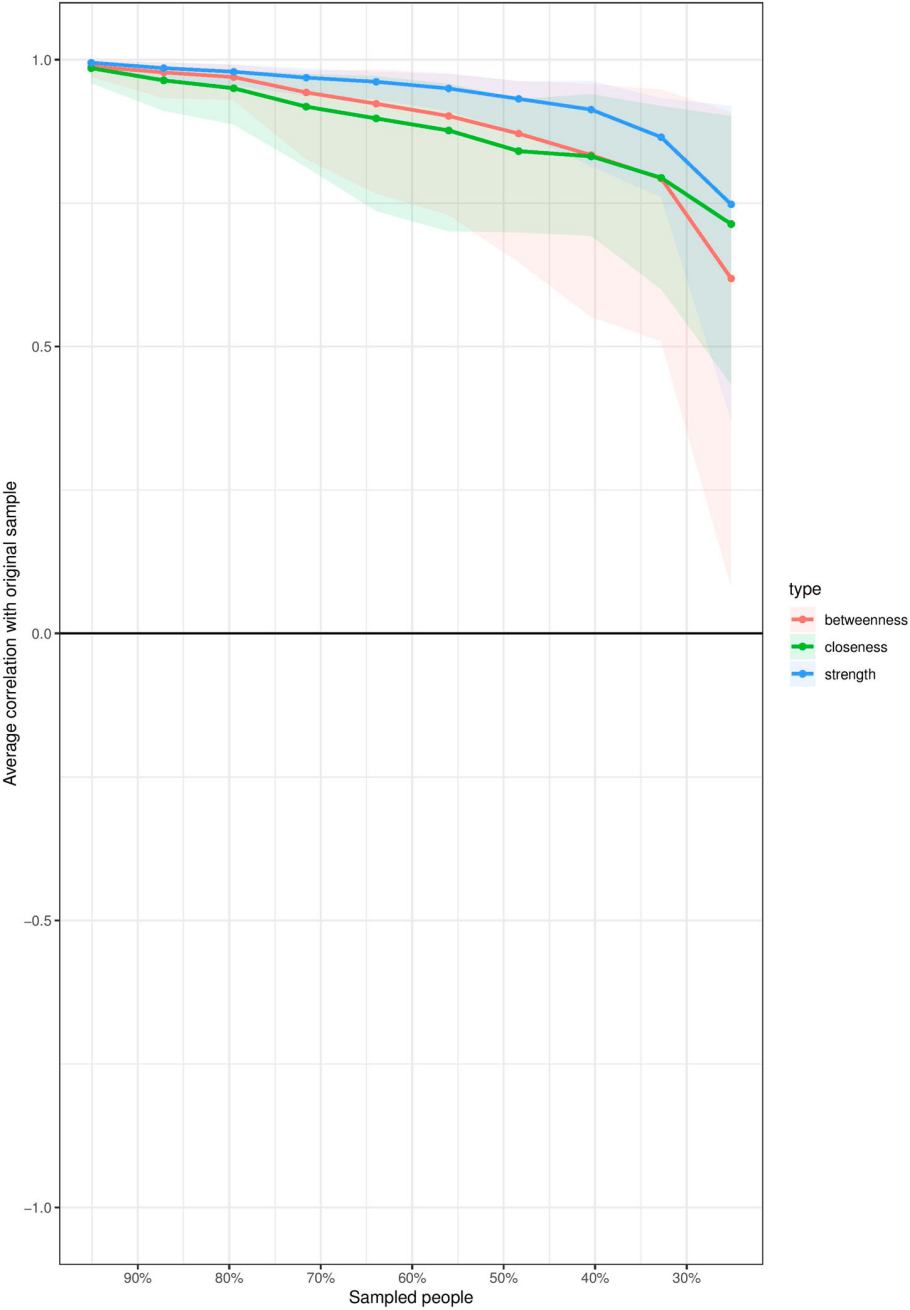
Stability of centrality indices.

Figure 2 shows that believing that organ donation is an act of helping others has the highest strength centrality score; whereas feelings of discomfort (uneasiness) about one’s organs being in someone else’s body has the highest closeness and betweenness centrality scores in the network. The strength centrality reflects the number of direct connections a particular node has, accounted for the strength of these connections. In our example, the belief that registering as an organ donor is an act of helping others is strongly and directly connected to many other nodes in the system relative to all other nodes. The closeness centrality indicates how close a particular node is to all other nodes in the network by incorporating indirect connections from that node. In our example, feeling uneasy regarding organ donation is well connected to many other nodes in the network – both directly and indirectly. Lastly, betweenness centrality indicates how often a node lies on the shortest path between other nodes. Nodes with high betweenness centrality are of particular importance because they connect variables that would otherwise be disconnected (a detailed account of network measures can be found in Hevey (2018)). After calculating the centrality measures, it is important to assess the stability of the estimated measures and the accuracy of edge weights (see Figure 3). We used bootstrapping methods to assess these properties (Hevey, 2018).

**Figure 4.**
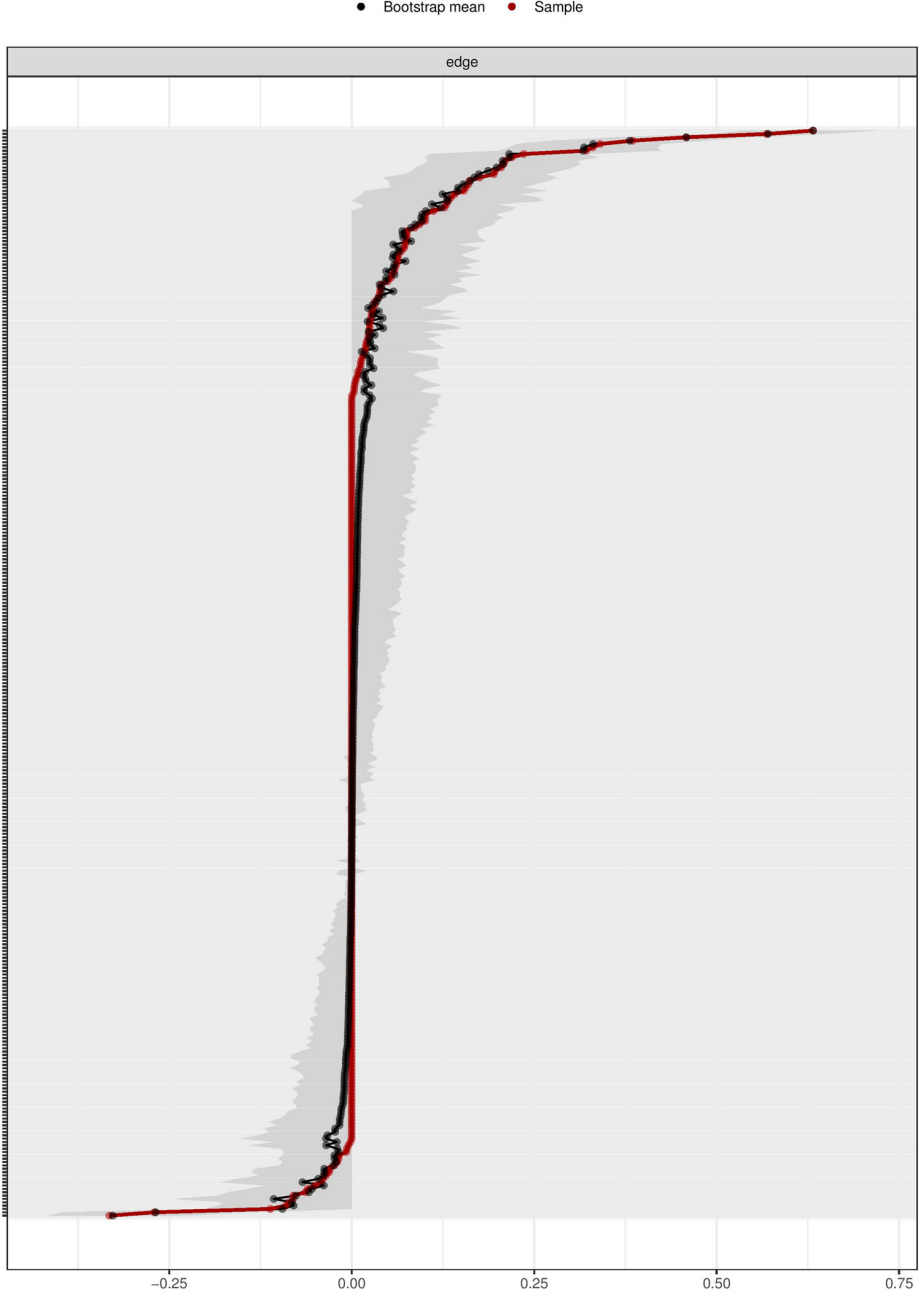
Accuracy of the edge-weight estimates and the associated 95% confidence intervals. Note: The red line indicates the sample values and the gray area the bootstrapped CIs. Each horizontal line represents one edge of the network, ordered from the edge with the highest edge-weight to the edge with the lowest edge-weight. The *y*-axis labels have been removed to avoid cluttering and the *x*-axis represents the scale of the edge weights.

In Figure 3, we see that most of the centrality indices are relatively stable. This is reflected in the strength of the correlations between the estimates of the subsamples and the original sample. As one can note, with subsets of 30% of the original sample (*x*-axis), all the estimates correlate more than 0.7 (*y*-axis). In Figure 4, we can see that the edge weights for most of the nodes in the network are close to the estimated bootstrap mean, indicating an acceptable level of accuracy.

In addition to the centrality scores, we calculated the shortest paths from all variables to the outcome variable by using Dijkstra’s algorithm^5^ (see Table 1) (Dijkstra, 1959; West, 2005). In Table 1, we can see that one’s belief that s/he has enough time to register as an organ donor has the shortest path to the outcome of interest. This indicates that this variable has the strongest association with one’s propensity of being registered as an organ donor compared to the other variables, which differs from traditional correlational analyses (Steenaart et al., 2018). However, such conclusion contains no information on the direction of such association, therefore it would be more meaningful to implement Dijkstra’s algorithm on negative and positive partial correlation matrices separately (see in the subsequent section).

**Table 1.** The shortest paths from each node to the outcome variable.

Nodes	Shortest path to registration status
Help	11.98
FamTalk1	5.71
FriendTalk2	20.95
TimeEnough	3.69
FriendTalk1	7.46
PosClosure	15.01
Reciprocity	30.30
FamDecision	13.39
Waste	7.55
DeformedPerson	18.31
ReciprocitySelf	28.33
Risk	21.62
DoctorsLife	20.46
ThinkDeath	17.08
Ownership	16.13
DeclareDead	23.57
FamTalk2	22.53
Uneasiness	8.97
KnowMore	12.48
ValueOrgans	21.21
Intact	13.86
KnowEnough	4.56
Mutilated	15.37
Allocation	16.22
DoctorsChoice	18.31

### Exploring network structures

**Figure 5.**
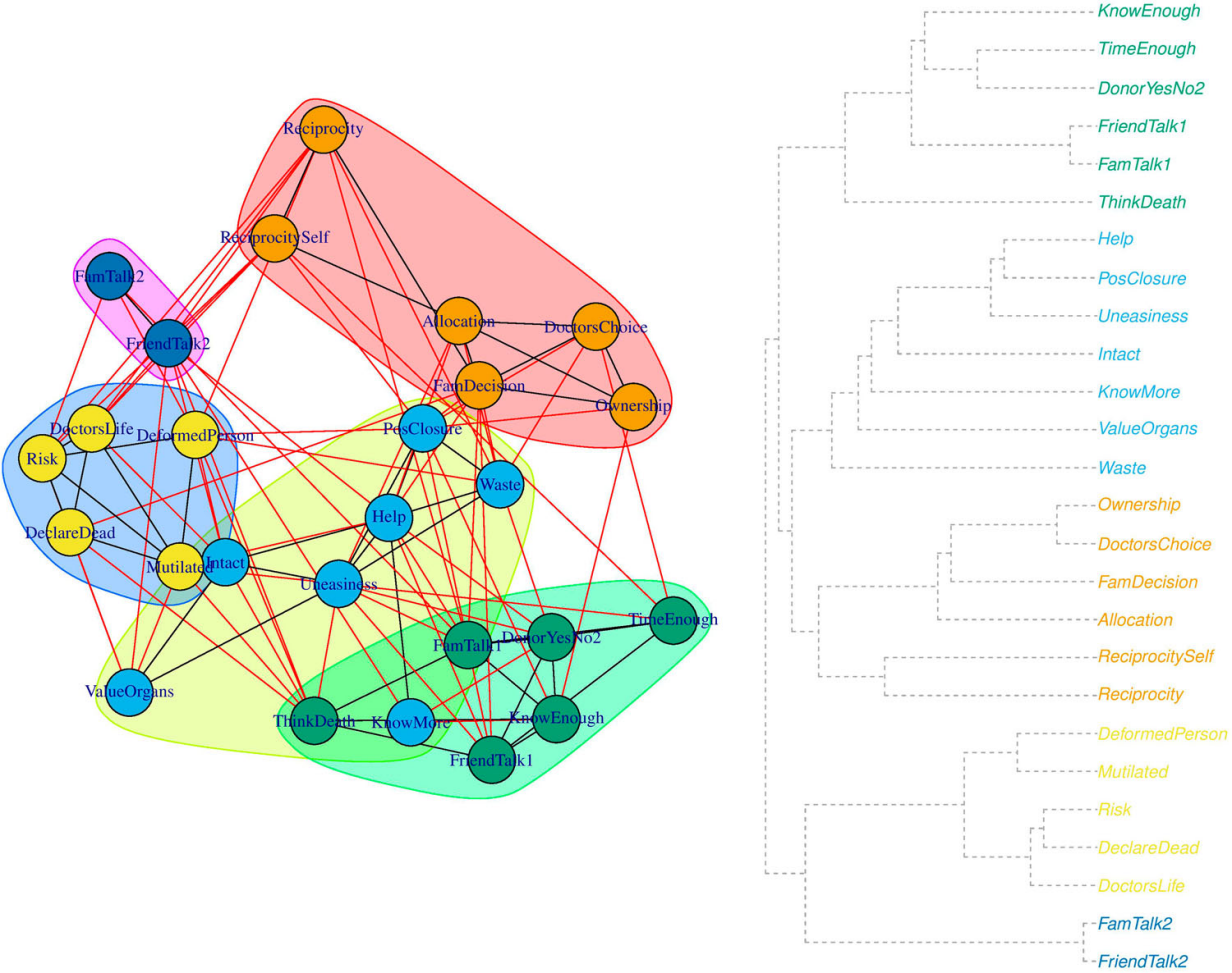
Hierarchical clustering of the network (on the left) and the respective dendrogram (on the right).

After examining the relationships between the nodes, we can examine the overall organization of the network. For example, we can investigate whether the variables tend to group together by conducting a cluster analysis. To achieve this we used a greedy agglomerative hierarchical clustering algorithm (Kolaczyk & Csárdi, 2014) (see Figure 5). The greedy algorithm calculates clusters by partitioning the network in such way that the resulting groups will contain structures that are stronger than expected to occur under the random assignment of edges (Kolaczyk & Csárdi, 2014; Newman, 2004). This type of analysis may help us examine whether items that belong to the same theoretical domain tend to group together. For example, one could check whether beliefs that are conducive to a particular behavior are clustered together. Subsequently, one may identify beliefs that have the strongest connections with all other beliefs in the cluster by calculating strength centrality within a given cluster.

In addition to examining clusters in the network, we may examine reinforcing and inhibiting structures in the network. It is important to note that the way in which networks are estimated may not actually reflect the ways in which beliefs are actually structured in people’s minds. Hence, before conducting such analysis and interpreting the results certain theoretical and methodological assumptions should be made explicit. The examples below are, therefore, just illustrations of the steps one can take were certain assumptions regarding the theory and methods correct.

Reinforcing structures include cyclic arrangements of variables that enable the circulation of a stimulus in such way that it incites a variable of interest. For example, to identify a reinforcing structure one can multiply the edge weights and if the product of the edge weights is positive then the component is reinforcing and its inhibiting if otherwise. It is extremely important to note, that reinforcing or inhibiting structures in a network imply a directed graph. More specifically, a cyclic structure means that a path from node A to node C can return to the node A. Because networks estimated in a cross-sectional data are undirected one should be cautious in interpreting such structures.

In general, we can differentiate two types of reinforcing and inhibiting structures – one related to the behavior of interest and one related to the constructs in the system. More specifically, the reinforcing and inhibiting structures in relation to the constructs reflect the processes that stimulate or inhibit the emergence of the constructs in the focal memory and its maintenance there. The reinforcing and inhibiting structures in relation to the behavior of interest reflect the structures that continuously enable or disable the accumulation of stimulation in the behavior of interest.

**Figure 6.**
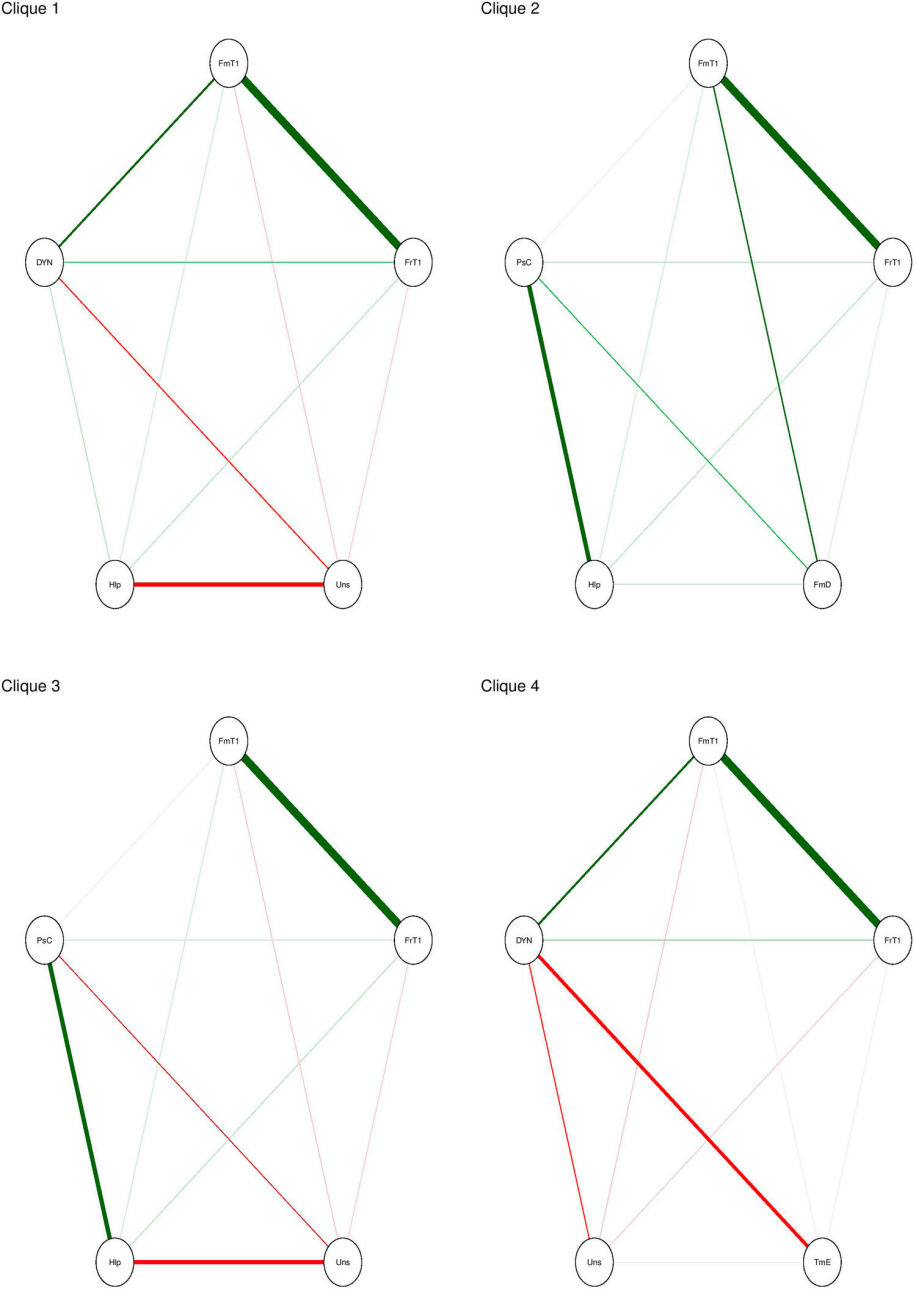
Four cliques of size five.

We examine such cyclic arrangements of variables by searching for triangles, four-cycles and cliques of *k* size in a given network. In our network, we have 104 triangles (cliques of size three), 38 four-cycles and 4 cliques of size five (see an example of four cycles in Figure 6).

In Figure 6, we can examine possible reinforcing structures. For example, in clique one, we notice that one’s feeling of easiness in talking to one’s family members is positively associated with the feeling of easiness in talking to friends which is positively associated with seeing the registration as organ donor as an act of helping others which in its turn is positively associated with talking to family members. Furthermore, all of the mentioned items are positively associated with one’s likelihood of registering as an organ donor. Postulating a dynamic causal process, we could say that by increasing one’s feeling of easiness in talking to family members about this topic will likely increase their feeling of easiness in talking to their friends, which will lead to perceiving the registration as an act of helping others. This in its turn increases the easiness of talking more to their families (e.g. because they see that their friends also share their perspective) and create a reinforcing loop where the importance of registering as an organ donor is being cultivated. In addition, we can see a reinforcing structure composed of three variables that include two negative associations (between uneasiness and registration status and uneasiness and help) and one positive (help and registration status). The latter cyclic triad enables continuous reinforcement of the registration status. It is worth noting that the supposition of causal processes without theoretical or empirical grounds is unwarranted.

### Exploring positive and negative correlations separately

**Figure 7.**
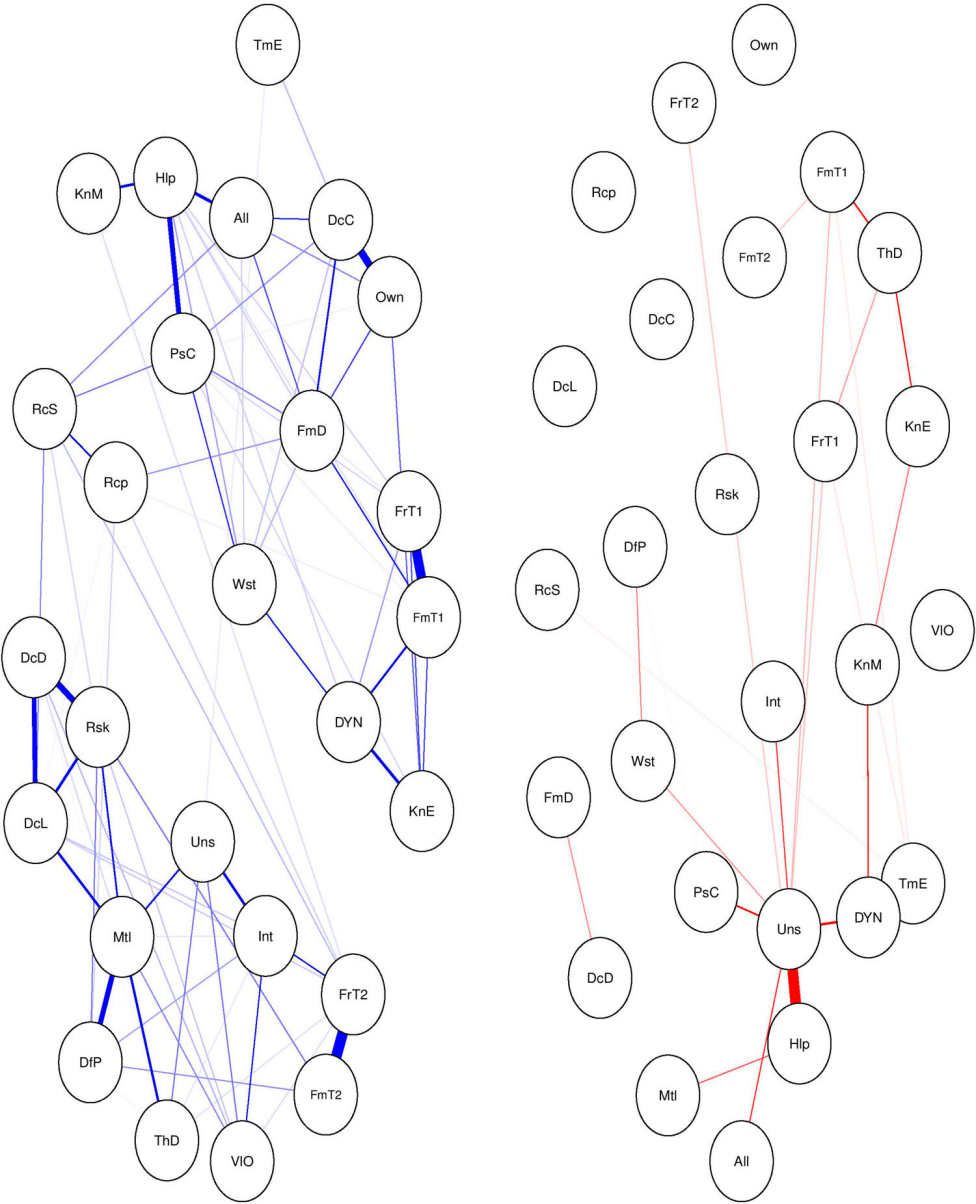
Network with the positive correlations on the left and network with the negative correlations on the right.

The centrality measures as well as Dijkstra’s algorithm take the absolute values of the edge weights in the calculations. This may be problematic because the negative and positive partial correlations convey different information and treating them similarly may result in different interpretations and conclusions. More specifically, the network with positive partial correlations reflects ‘stimulating’ effects (i.e. increase in one or more variables is associated with an increase in the other variables in the network). In contrast, the network with negative correlations reflects a type of ‘wave effects’ (under certain assumptions) – an increase in one variable is associated with a decrease in another variable, which in its turn is associated with an increase in the third variable. In addition, looking at reinforcing and inhibiting structures in networks with only positive and negative correlations may increase the visibility of such structures, particularly in dense networks with many variables. Therefore, separating networks of positive and negative correlations may be more appropriate for calculating and interpreting the centrality measures as well as for the examination of reinforcing and inhibiting structures. We illustrate this approach in Figure 7.

**Figure 8.**
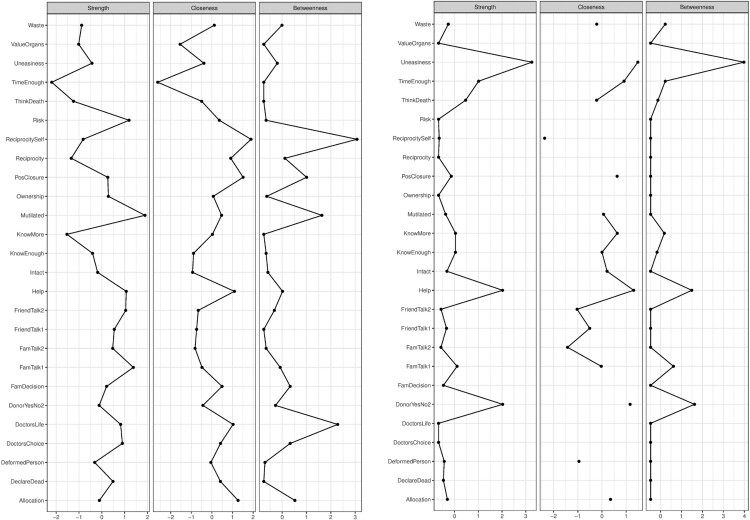
Centrality scores of each node for networks with positive correlations (on the left) and negative correlations (on the right).

Now we can explore the relative importance of the nodes in networks representing positive and negative partial correlations. We calculated the same centrality measures as in the case of the entire network (see Figure 8).

In the network with only positive partial correlations, we can see that ‘concerns about mutilation’ has the highest strength centrality score and ‘reciprocity’ has the highest closeness and betweenness centrality score. In other words, one’s fear for being mutilated in case of being an organ donor is strongly and directly connected to many other variables in the network. This means that decreasing one’s concerns about mutilation will likely decrease the scores of many other variables in the network. The ‘reciprocity’ scores indicate that one’s belief that registering as an organ donor makes oneself more deserving to receive an organ, in case they need one, more often mediates the relationship between the other variables in the network with negative partial correlations. This means that increasing one’s belief of reciprocity may greatly affect variables that are not directly linked to each other.

It is imperative to note that splitting networks based on negative and positive correlations can be problematic because (among other reasons) it may distort the multivariate structure of the network and results in inaccurate conclusions. However, on the other hand calculating centrality scores on the network with both positive and negative partial correlations may also be inaccurate. Therefore, we suggest to use this approach with caution and perhaps apply it in an exploratory way. In addition, it is important to note that the utility of centrality metrics in psychological networks is a subject of an ongoing debate in the field (Bastiaansen et al., 2019; Bringmann et al., 2019). It is important that in each use case one provides a rationale for a particular choice of the used centrality metrics and the assumptions that underly these metrics (Bringmann et al., 2019).

Subsequently, we can recalculate the shortest paths for each network separately (see Table 2). In Table 2, we can see that the calculated shortest paths are different in positive and negative partial correlation matrices. In the positive correlation matrix, one’s belief that s/he has enough knowledge to make a decision about organ donation has the strongest association with one’s propensity to register as an organ donor. Whereas, similar to the full network ‘one’s belief that s/he has enough time to register’ has the shortest path to the outcome of interest in the network with negative partial correlations. Additionally, it could be interesting to look for structural components in a network that may have inhibiting^6^ effects.

**Table 2.** The shortest paths from all nodes to the outcome variable in networks with positive and negative partial correlations.

Nodes	Shortest path to registration status	
	Positive partial correlations	Negative partial correlations
Help	19.51	11.98
FamTalk1	5.71	50.92
FriendTalk1	7.46	59.03
PosClosure	16.48	20.07
Reciprocity	30.30	–
Waste	7.55	41.26
FamDecision	13.39	–
Risk	49.30	–
DoctorsLife	44.49	–
Ownership	16.13	–
KnowEnough	4.56	36.38
DeformedPerson	52.52	66.78
FriendTalk2	56.17	67.46
ReciprocitySelf	29.80	228.84
Allocation	23.75	24.71
ThinkDeath	54.72	48.90
DeclareDead	47.61	–
ValueOrgans	65.38	–
Intact	60.87	28.59
DoctorsChoice	18.31	–
KnowMore	24.27	12.48
Mutilated	49.58	31.64
TimeEnough	43.99	3.69
FamTalk2	57.75	108.46
Uneasiness	55.98	8.97

It is important to note that the structural characteristics in the network discussed so far do not express much about structural features that are unique to a given behavior. This is because by including the behavioral variable as a component of a system we remove its variance when estimating the partial correlations between other components of a system. In the above-mentioned example of a cyclic structure between one’s feeling of easiness in talking to one’s family members, feeling of easiness in talking to friends, seeing registration as organ donor as an act of helping others and talking to family members are adjusted for one’s status of being registered as an organ donor. Therefore, this cyclic structure is presumably present regardless of one’s registration status. If one is interested in structural components that are unique to a given behavior, one ought to deploy a different analytical strategy as we discuss later in this paper.

## Behavior as an emergent phenomenon

Another way of conceptualizing the behavior of interest is to view it as emerging from the system of its determinants. In this framework, specific patterns of related determinants (e.g. beliefs) give rise to the behavior of interest through spreading activation (Peters & Crutzen, 2017). These patterns of related determinants can be construed as mental representations, which are cognitive structures that reflect accumulated knowledge and experience on which cognitive processes operate (Gawronski & Payne, 2010). Mental representations are often associated with such constructs as concepts, schemas, impressions, attitudes and stereotypes. Spreading activation models describe how memory is organized and how learning and retrieval processes take place. Once activated, mental representations emerge in the focal memory and trigger the activation of other related concepts through associative pathways. After sufficient activation level is achieved in these related concepts, they also reach focal memory and emerge in consciousness. This activation mechanism follows a type of a chain reaction that starts from one node and spreads to others (Gawronski & Payne, 2010). The activation pattern can also occur in parallel (i.e. simultaneous activation). Considering the interrelationships between constructs in the boundaries of the conceptualizations of spreading activation, we can view constructs as not causing each other in a literal sense but rather causing/facilitating each other’s emergence in focal memory. The longer the co-activated concepts remain in the focal memory the stronger the associations between them become which leads to changes in the network of long-term memory. These changes in long-term memory are linked to associative learning and can explain the increased accessibility of the frequently activated constructs (also referred to as chronically activated concepts).

The mentioned co-activation of concepts may occur not only due to intra-individual processes (in someone’s brain) but also because of various external factors (e.g. reinforcement by others, environmental cues). To illustrate this, consider the process of reinforcement learning that posits that people change their behavior in order to maximize the associated rewards and minimize the punishments. Such external reinforcements and punishments are likely to determine the underlying structure of the observed network of the mental models. For example, these external reinforcing or punitive processes may determine the cyclic structures we discussed in the previous section.

**Figure 9.**
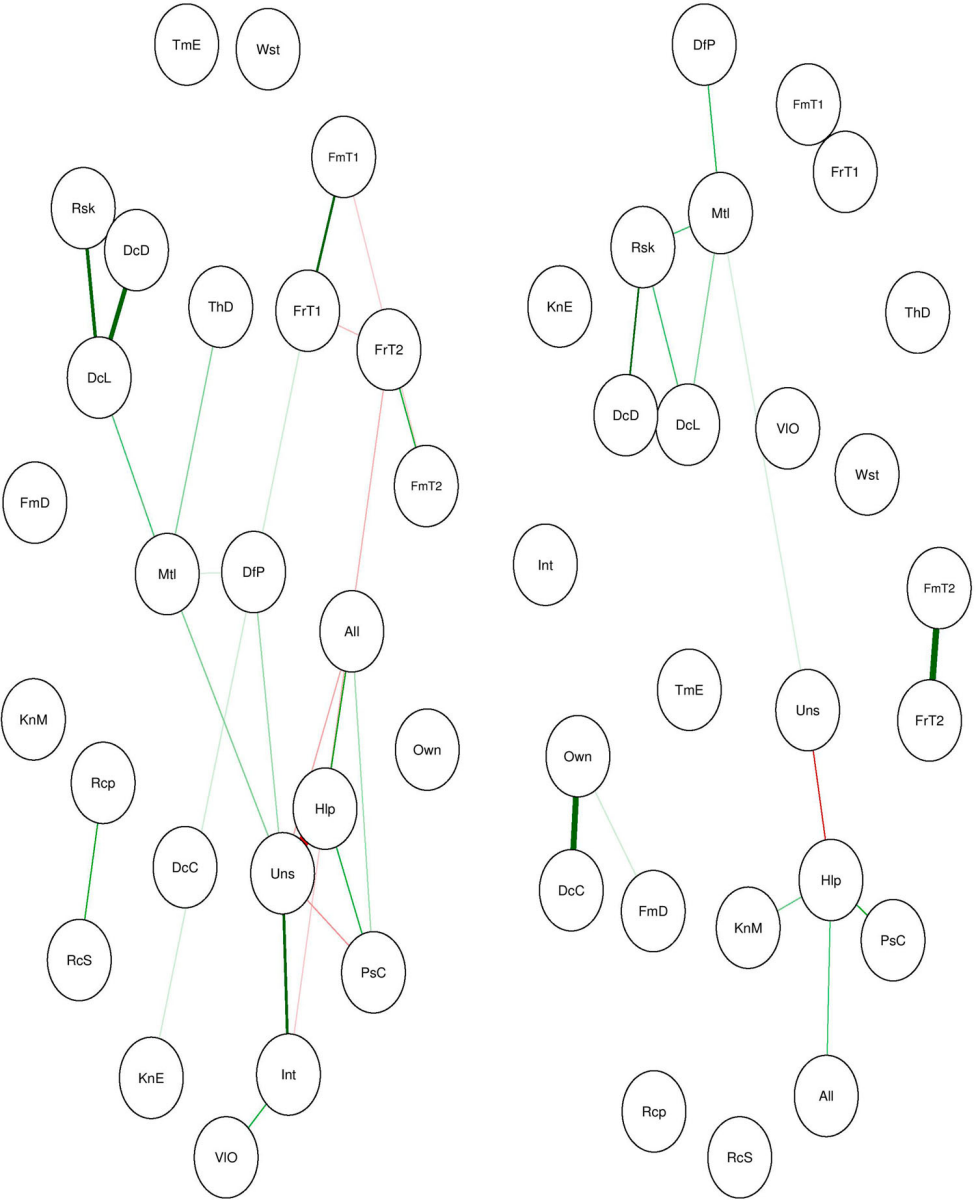
Estimated network of registered organ donors (on the left) and non-registered participants (on the right).

The conceptualization of behavior as an emergent phenomenon entails an estimation of two separate networks – one where the behavior of interest is present and one where it is absent. This way we have the opportunity to look for distinct network features (network topologies) that define or characterize the behavior of interest. To illustrate this approach, we split our data based on the registration status (i.e. registered and not registered). After splitting the data, we simultaneously estimated the corresponding networks by using recently introduced Fused Graphical Lasso (FGL) method (Costantini et al., 2019) (see Figure 9).

**Figure 10.**
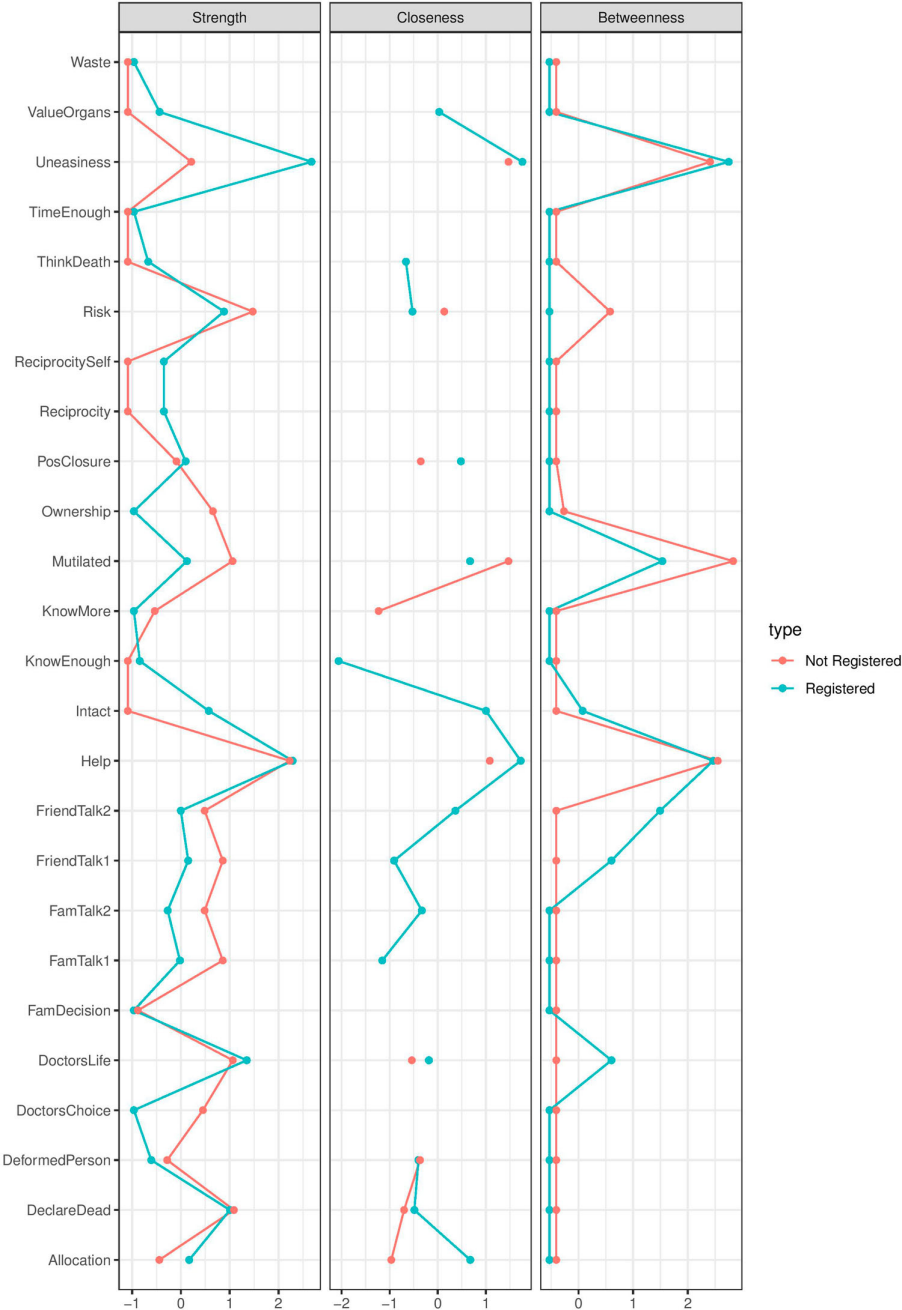
Centrality plot between the groups.

The estimated networks differ in their overall structure. The network of participants who are registered as organ donors seem to have larger densely connected components compared to the group who are not registered as organ donors. The content of the components is also different between groups (see Figure 10). By contrasting these two networks one can find distinct structural features that may explain the behavior of interest.

In addition to visual inspection, one could conduct a Network Comparison Test (NCT) to statistically test whether the various structural components are statistically different between the networks (van Borkulo et al., 2016). The NCT compares two networks based on network structure, global strength, and edge measures.

The network comparison test indicates that there are no statistically significant differences in network structure (*M* ^7^ = 0.53, *p* = 0.05) and global strength (*S* ^8^ = 5.82, *p* = 0.133) between the networks of people who are registered as organ donors and those who are not. One should note that the statistical significance of the test statistic of the NCT like other statistical tests is a function of the sample size and the size of the difference (van Borkulo et al., 2016). In our case, the sample size was quite small and imbalanced (*N*_registered _= 93, *N*_not-registered = _273). A priori sample size calculations as well as power analysis is of great importance before substantive conclusions can be drawn and also receives increased attention within network analyses (Epskamp & Fried, 2018).

Similar to the steps described in the prior section, one can examine the relative importance of each node, the overall organization of the network as well as explore reinforcing and inhibiting structural components in the networks and draw comparisons between the results.

## Ethics statement

The paper uses secondary data of the study by Steenaart, Crutzen, & de Vries, 2018. The study was approved by the ethics committee of the Faculty of Health, Medicine and Life Sciences on February 14, 2017 (reference number: FHMLREC/2017/01).

## Discussion

**Figure 11.**
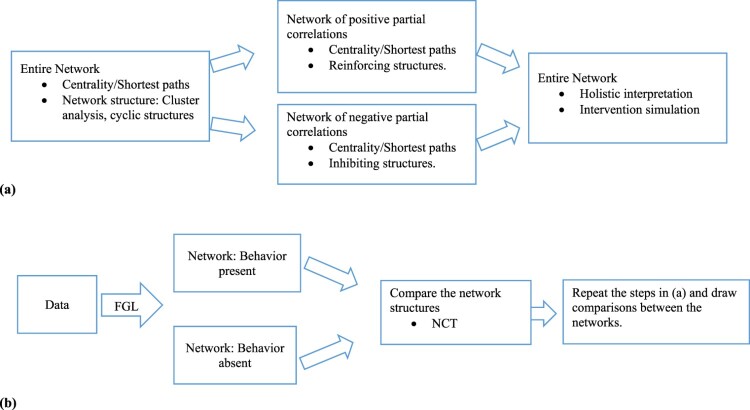
Two approaches of incorporating observable behavioral variable in a system: (a) behavior as a constituent in a system and (b) behavior as an emergent phenomenon.

The network approach provides a powerful tool and a framework for analyzing psychological variables in a system. Previous applications of a network approach to psychological variables lacked a discussion on what the system of psychological variables represents and what the interrelationships between the variables mean (Brandt et al., 2018; Dalege et al., 2016; Hevey, 2018; Rucci et al., 2018; Schmittmann et al., 2013; van Zyl, 2018). In addition, previous work has largely focused on node level measures thus somewhat neglecting the examination of the structural components of the overall system. In this paper, we discussed the system of psychological variables in the realms of theories on spreading activation in social cognition and illustrated two approaches of incorporating an observable behavioral variable in a system (see a schematic representation in Figure 11).

The first approach, commonly used in prior applications, treats the observable behavioral variable as a constituent in the system. In this approach, we suggest that one can conceptualize the system of psychological variables as mental representations. Furthermore we view the interrelationships between psychological variables as causing or inhibiting each other’s emergence in the focal memory on which cognitive processes operate (Gawronski & Payne, 2010). Following such conceptualization of the system, one can construe the behavioral variable as a component in a system these mental representations directly or indirectly stimulate. The first approach permits investigation of learning processes and behavioral feedback loops, however, it does not allow exploration of structural features of a system unique to a behavior of interest (topology). In the second approach, we view the behavior of interest as emergent phenomenon that appears from a system of psychological variables. As in the first approach, the interrelations between the variables are considered in the scope of theories of spreading activation. More specifically, the concepts are viewed as causing each other’s emergence in focal memory through activation pathways (Gawronski & Payne, 2010). With the second approach, one can search for distinct patterns of these interlinked concepts that presumably give rise to the behavior of interest. In this regard, we illustrate the use of cluster analysis (e.g. hierarchical clustering) and examination of cyclic structures (e.g. triangles, and cliques of *k* size) for exploring network topologies. It is important to note that there are different clustering techniques and the researchers ought to select an algorithm that best fits the specifics of their inquiry (Kolaczyk & Csárdi, 2014).

In addition to providing a conceptual framework for a system of psychological variables and the treatment of observable outcome variables, we provided a rationale for separating networks of positive and negative partial correlations during the calculations of centrality measures as well as shortest paths to focal variable. We also illustrated that examining the positive and negative partial correlation networks separately may ease the exploration of reinforcing and inhibiting structures in a given network particularly in a network with many variables. However, in order to have a holistic understanding of the potential impact of intervention one ought to eventually go back to the entire network and see how a hypothetical intervention would affect the entire system given positive and negative relationships in the network. One could also examine the potential impact of interventions by using network simulations (Borsboom & Cramer, 2013). Network simulations may illustrate how network will change under hypothetical intervention scenarios.

It is worth mentioning that the treatment of the outcome of interest as an emergent phenomenon comes with a price. The estimation of two separate networks requires larger sample size in each group (i.e. with and without the behavior of interest). The sample size is also imperative for statistical comparison tests such as NCT. The importance of sample size calculations and ways to tackle the problem of accuracy under sampling variation have recently been introduced for network analyses (Epskamp & Fried, 2018). Additionally, the analysis of a subgroup of sample may result in Berkson’s bias and one shall consider additional analytical steps (see De Ron, Fried, & Epskamp, 2019).

Lastly, it is important to acknowledge the limitations related to cross-sectional data and highlight the importance of using longitudinal network models to incorporate dynamic processes of behavior change. Cross-sectional network analysis does not allow extensive topological examination of the system (e.g. feedback loops). Longitudinal network analysis, on the other hand, reveal important structural characteristics of the system and show dynamic processes involved in the system. The analysis of these dynamic processes in a longitudinal network may shed light on how relationships between variables and network structure evolve over time. Such longitudinal models include Temporal Exponential Random Graph (TERGM) models and Graphical Vector Autoregressive Models (Graphical VAR) (Epskamp et al., 2018).

In general, empirical network analysis may provide important insights into the systems of psychological variables and shows promising results in terms of replicability of findings (Borsboom et al., 2017). Analytical methods in this field are being developed and refined continuously.

We hope that this paper will provide researchers with guidance on network analysis of psychological variables. In addition, we anticipate that it will facilitate a discussion on the conceptualizations of networks of psychological variables, which will guide the analysis and the interpretation of node level interactions as well as network level structures.

## Supplementary Material

Supplimental_Materials.docx

## Notes

1 We use a broad definition of health behavior as ‘any activity undertaken for the purpose of preventing or detecting disease or for improving health and well-being’ (Conner and Norman, 2005)

2 It is worth noting that the contrast between latent variable approach and network approach is ingrained in the current academic debate. We do not reflect on this debate because it goes beyond the scope of this paper, however we refer an interested reader to Bringmann and Eronen (2018).

3 In this paper, we use the terms system and networks interchangeably.

4 Because of the illustrative nature of this paper, we only used the items pertaining to beliefs, and registration status regarding organ donation. The non-identifiable data, syntax, and output of the analyses are available at https://osf.io/4m67q/. These efforts are taken to acknowledge a recent call for full disclosure to maximize scrutiny, foster accurate replication, and facilitate future data syntheses (e.g., meta-analyses) (Crutzen, Peters, & Abraham, 2012; Peters, Abraham, & Crutzen, 2012). We estimated the network following the steps described in Hevey (2018) and the R code for the present analysis can be found in the supplementary materials.

5 Note that before using Dijkstra’s algorithm one ought to invert the partial correlation coefficients so the strongest connections will be represented with smaller numbers (shorter distance) and the smallest correlations with larger (longer distance).

6 We did not detect any inhibiting cyclic structures in the network.

7 M is the value of the maximum difference in edge weights of the observed networks.

8 S is the difference in global strength between the networks of the observed data sets.
