# Supplementary material for: Network approach in health behavior research: how can we explore new questions?
Source: Health Psychol Behav Med. 2019 Nov 5;7(1):362–84. doi: 10.1080/21642850.2019.1682587 (PMC8114378; doi:10.1080/21642850.2019.1682587)
Supplement: Supplimental_Materials.docx [file RHPB_A_1682587_SM4528.docx]

**Annexes**

**Annex 1:** List of variables and the respective abbreviations

| **Item** | **Variable Name** | **Abbreviation** |
| --- | --- | --- |
| 1. I have enough knowledge to make a decision about organ donation | KnowEnough | KnE |
| 1. I would like to know more about organ donation and –registration | KnowMore | KnM |
| 1. I have talked about organ donation with family members | FamTalk1 | FmT1 |
| 1. I find it hard to discuss organ donation with family members | FamTalk2 | FmT2 |
| 1. I have talked about organ donation with friends | FriendTalk1 | FrT1 |
| 1. I find it hard to discuss organ donation with friends | FriendTalk2 | FrT2 |
| 1. I rather not think about death | ThinkDeath | ThD |
| 1. I still have enough time to register | TimeEnough | TmE |
| 1. The idea of my organs being in someone else’s body gives me a feeling of discomfort | Uneassiness | Uns |
| 1. Certain organs have an important value to me | ValueOrgans | VIO |
| 1. When I die, I don’t want my organs to go to waste | Waste | Wst |
| 1. When I die, my body needs to be intact for afterlife | Intact | Int |
| 1. Some people are more deserving to receive an organ than others | Reciprocity | Rcp |
| 1. If I register my decision, I prevent my family from having to make a difficult decision when I would die | FamDecision | FmD |
| 1. If I register my decision, medical professionals will honor my wishes | DoctorsChoice | DcC |
| 1. If I register my decision, I know what happens with my body when I would die | Ownership | Own |
| 1. If I am an organ donor, I am happy I can help people in need | Help | Hlp |
| 1. If I am an organ donor, I am afraid my family and friends see me as a deformed person because my organs were removed | DeformedPerson | DfP |
| 1. If I am an organ donor, I can find a sense of positive closure | PosClosure | PsC |
| 1. If I am an organ donor, I run the risk of my organs being taken out before I died | Risk | Rsk |
| 1. If I am an organ donor, I am afraid my body will be mutilated | Mutilated | Mtl |
| 1. If I am an organ donor, medical professionals will choose the life of a patient who needs an organ over mine | DoctorsLife | DcD |
| 1. If I am an organ donor, I am more deserving of receiving an organ in case I need one | ReciprocitySelf | RcS |
| 1. If I am an organ donor, my organs will be allocated to patients in an ethical manner | Allocation | All |
| 1. If I am an organ donor, I run the risk of being declared dead too soon | DeclareDead | DcL |
